# Non‐traditional Dementia Caregivers: Does a more distant relation diminish burden?

**DOI:** 10.1002/alz.087598

**Published:** 2025-01-09

**Authors:** Amanda N. Leggett, Jonathan Troost, Sophia Tsuker, Jennifer A. Miner, Noelle E. Carlozzi

**Affiliations:** ^1^ Wayne State University, Detroit, MI USA; ^2^ University of Michigan, Ann Arbor, MI USA

## Abstract

**Background:**

Examination of family caregiving and the stress process has focused on a “primary” caregiver (e.g., spouse, adult child) at the exclusion of other members of the caregiving network. Yet “non‐traditional” (e.g., grandchild, friend) caregivers are increasingly providing substantial caregiving support. This study explores whether non‐traditional dementia caregivers vary from traditional caregivers in terms of caregiving approaches and outcomes.

**Method:**

Participants included 200 family caregivers (n = 156 traditional and n = 54 non‐traditional) for a person living with dementia from the Style Measure Study. Descriptive statistics and t‐tests were run to examine differences between traditional and non‐traditional caregivers. Linear regressions were run adjusting for caregiver demographics and the care context with caregiver type predicting use of dementia management strategies (criticism, encouragement, active management), Neuro‐QOL measures of caregiver strain, caregiver‐specific anxiety, feeling trapped, and positive affect, PROMIS measures of sleep‐related impairment, fatigue and pain intensity, and caregiver readiness.

**Result:**

Non‐traditional caregivers were significantly younger, more likely to be Black, had higher levels of financial difficulty, had provided care for less time, and reported significantly greater pain intensity and significantly lower levels of caregiver readiness. In adjusted linear regressions however, caregiver type only predicted use of active management care strategies; traditional caregivers used more active management than non‐traditional caregivers (B = ‐2.24, *p*<.05).

**Conclusion:**

Non‐traditional caregivers are notably distinct from their traditional counterparts demographically, yet non‐traditional status generally did not predict differences in caregiving outcomes. Future research should prioritize inclusion of diverse caregivers to represent the broader caregiver population and recognize that their care needs are just as extensive as traditional caregivers.

